# Metagenomic Characterization Reveals Pronounced Seasonality in the Diversity and Structure of the Phyllosphere Bacterial Community in a Mediterranean Ecosystem

**DOI:** 10.3390/microorganisms7110518

**Published:** 2019-11-01

**Authors:** Despoina Vokou, Savvas Genitsaris, Katerina Karamanoli, Katerina Vareli, Marina Zachari, Despoina Voggoli, Nikolaos Monokrousos, John Maxwell Halley, Ioannis Sainis

**Affiliations:** 1 Department of Ecology, School of Biology, Aristotle University of Thessaloniki, 54124 Thessaloniki, Greece; 2 School of Economics, Business Administration and Legal Studies, International Hellenic University, 57001 Thermi, Greece; s.genitsaris@ihu.edu.gr; 3 School of Agriculture, Aristotle University of Thessaloniki, 54124 Thessaloniki, Greece; katkar@agro.auth.gr; 4 Department of Biological Applications and Technology, University of Ioannina, 45110 Ioannina, Greece; kvareli@cc.uoi.gr (K.V.); marou92@hotmail.gr (M.Z.); d_voggoli@hotmail.com (D.V.); jhalley@cc.uoi.gr (J.M.H.); isainis@cc.uoi.gr (I.S.); 5 Department of Soil Science of Athens, Hellenic Agricultural Organization-Demeter, Institute of Soil and Water Resources, 14123 Lykovrisi, Greece; nmonokro@bio.auth.gr

**Keywords:** air, bacterial colonization, epiphytic bacteria, high-throughput sequencing, generalists, specialists, 16S rRNA gene

## Abstract

We explore how the phyllosphere microbial community responds to a very seasonal environment such as the Mediterranean. For this, we studied the epiphytic bacterial community of a Mediterranean ecosystem in summer and winter, expecting to detect seasonal differences at their maximum. With high-throughput sequencing (HTS), we detected the operational taxonomic units (OTUs) present in the phyllosphere and also in the surrounding air. The epiphytic community is approximately five orders of magnitude denser than the airborne one and is made almost exclusively by habitat specialists. The two communities differ considerably but Proteobacteria and Actinobacteria are dominant in both. Of the five most abundant phyllosphere OTUs, two were closely related to *Sphingomonas* strains, one to *Methylobacterium* and the other two to Rhizobiales and Burkholderiales. We found the epiphytic community to become much richer, more distinct, even and diverse, denser and more connected in summer. In contrast, there was no difference in the level of bacterial colonization of the phyllosphere between the two seasons, although there were seasonal differences for individual taxonomic groups: Firmicutes, Gemmatimonadetes and Chlroroflexi had a higher participation in summer, whereas the major Proteobacteria classes presented reverse patterns, with Betaproteobacteria increasing in summer at the expense of the prominent Alphaproteobacteria.

## 1. Introduction

Life on leaf surfaces that are exposed to extreme variations in meteorological and other environmental factors looks like the life of a fugitive. However, existing evidence suggests that microbial communities are well established, that only a subset of the air-introduced taxa can colonize them and that leaves are not passive acceptors of microbes deposited from the air [1,2,3]. There is also growing evidence indicating the involvement of leaf bacteria in important interactions that may affect plant fitness [4,5] and quality and the production of crop plants [6,7,8], and even in processes at a global scale such as nitrogen fixation [9]. 

Phyllospere microorganisms can arrive as bioaerosols, via rainfall or irrigation water [10,11], by animals [12], particularly herbivorous insects [10], and also as colonizers of other plant parts at earlier stages of the plant ontogeny [10,13]. They may be of plant, animal, water or soil sources, but the relative contribution of each source is still unclear [14]. Air is an important medium for their transfer and further deposition on leaf surfaces. Models of atmospheric circulation suggest that particles of the size of bacterial cells can move readily between continents within a year [15], but dispersal limitations may make local sources more important than distant ones for the microbial colonization of the phyllosphere [10]. In a study using high-throughput sequencing (HTS), it was estimated that up to 50% of airborne bacteria in downwind air samples were of local plant origin [16].

Culture-independent methods have shown that leaf microbial communities are much more diverse than previously thought and provided insight on the composition, the physiological aspects and the niches of the leaf-associated microorganisms [17,18]. New tools such as HTS enabled studies dealing with the structure and variation of the epiphytic bacterial community, and its functions and interactions with biotic and abiotic factors, to rapidly increase [5,14,19]. However, phyllosphere studies concern mostly man-made environments [20], crops or other plants of economic importance [18] and their pathogens [21], and usually one [22] or a few plant species [23]. Few have examined epiphytic communities in natural environments, at the ecosystem level, and even fewer examined their features in more than one time slots [2,24] and/or in relation to the air inoculum [25]. 

Studies at the ecosystem level can provide a better understanding of the factors that determine phyllosphere colonization and expand our knowledge regarding complex dynamics at play. Published studies that focus on the structure of the epiphytic bacterial community concern the tropical [4,9], the Atlantic [26,27], the temperate [28] and other forests [29] or ecosystem types [3,30]. To our knowledge, very few studies deal with the epiphytic bacterial communities from areas with typical Mediterranean climate—those that do include the study by Peñuelas et al. [31] and those exploring the epiphytic microbial community of an ecosystem at Halkidiki, northern Greece [1,32,33,34]. With culture-dependent methods, it was found that phyllosphere bacterial populations of the latter ecosystem are lognormally distributed, with their size ranging from non-detectable to a maximum of ~10^7^ CFU g^−1^ [32] and that phyllosphere colonization is influenced primarily by the leaf water content followed by the phosphorus content and the thickness of the adaxial epidermis [33]. Using first-generation molecular techniques, denaturing gradient gel electrophoresis (DGGE), in particular, Vokou et al. [1] evidenced differences between the airborne and the epiphytic communities, prominence of bacteria associated with only one plant species and a frequent occurrence of lactic acid bacteria on leaves. 

An important question regarding the microbial community of the phyllosphere, which is exposed to large variations in meteorological parameters, is how it behaves in response to a very seasonal environment such as the Mediterranean. Does it keep its structure with only minor fluctuations, or does it change overall? To answer to this question, we focused on bacteria and studied their phyllosphere community in summer and winter. These are the two most contrasted seasons of the Mediterranean climate, characterized by the stress factors of drought and low temperatures, respectively. By studying the microbial community at these times, we expect to detect the seasonal differences at their maximum. We performed this study in the same Mediterranean ecosystem mentioned above, at Halkidiki, Greece, sampling from the same species and in the same way as before [1,32,33,34,35]. Using HTS, we aimed at identifying the microbial taxa present, estimate their contribution to the epiphytic microbial community, detect their dominant life strategies (specialists, generalists), and further examine if seasonal changes are only quantitative (in abundance), or whether they are of more fundamental character associated with differences in the number, identity, occurrence, and dominant functional traits of the taxa present. Considering the air as a most important medium for microbial transfer [2], we also examined how much the established community in the phyllosphere deviates at the time of sampling from the transient air community. These explorations allow a better understanding of the structure of the microbial community of the Mediterranean phyllosphere, of the dominant players and their strategies, and also of their responses to the marked seasonality of the Mediterranean environment.

## 2. Materials and Methods

### 2.1. Study Site

The study site is in close proximity to the sea, at Halkidiki, in northern Greece (40°09 N, 23°54 E), an area with a Mediterranean-type climate; July is the hottest month of the year. To assess the phyllosphere and airborne microbial communities, we sampled from an area of less than 5 ha, in summer (July) and winter (January). In both cases, sampling started approximately 1.5 h after dawn on rainless and practically windless days and lasted for approximately 3 hrs. We collected weather data for the days of sampling from the weather station of Neos Marmaras, which is the nearest station to our sampling site, at a distance of 13 km on a straight line. We also took measurements of temperature and wind speed on the spot, at the end of sampling (Supplement Table S1). 

### 2.2. Sampling and Sample Processing

To assess the epiphytic microbial community of this Mediterranean ecosystem, we took leaf samples from nine perennial plant species that differ in plant habit and associated leaf traits [35] and are representative of the local vegetation and the phyllosphere habitats that are available for microbial growth. These are the evergreen sclerophyllous *Arbutus unedo, Myrtus communis, Phillyrea latifolia, Pistacia lentiscus* and *Quercus coccifera*, the seasonal dimorphic *Cistus incanus* and *Lavandula stoechas,* and the herbaceous *Calamintha nepeta* and *Melissa officinalis*. The sampled area consisted of a flat land, at the mouth of a small torrent that is dry in summer, and of two low-hill slopes at each side: the more mesic and densely covered NE facing slope, dominated by evergreen sclerophyllous species, and the more xeric SW slope, dominated by seasonally dimorphic species. The herbaceous species were collected from the flat land.

For each selected species, at random, we collected mature leaves from five mature individuals. Approximately 0.3 g of the leaves of each sampled individual was weighed and put in 1.2 mL of sterile phosphate buffer (1XPBS: 137 mM NaCl, 10 mM phosphate, 2.7 mM KCl, pH 7.4). Samples were then sonicated in an ultrasonic cleaner for 10 min with the temperature of water not exceeding 20 °C. Buffer suspensions were centrifuged (3-30K Sigma GmbH, Osterode am Harz, Germany) at 9500 rpm, at 4 °C, for 20 min. Pellets of the same species were kept at −20 °C and analyzed as one sample in DNA extraction.

Air sampling took place at the flat land, close to the populations of the herbaceous species, at almost equal distance from the populations of the evergreen schelophyllous and the seasonally dimorphic species that were sampled. For this, we used an Andersen six-stage microbial impaction sampler (Andersen 2000 Inc., Atlanta, GA, USA) deployed on a tripod at a height of 1.5 m above ground. Air sampling was carried out twice for each sampling day, once in the beginning and once at the end of the phyllosphere sampling. Airborne bacteria were collected by means of vacuum filtration for 15 min at a flow rate of 28.3 L min^−1^ on sterile cellulose filters (0.22 μm pore size, 90 mm diameter, MF-Millipore; Merck KGaA, Darmstadt, Germany) that were placed on top of the Andersen plates. These filters were then aseptically put into test tubes containing 10 mL sterile phosphate buffer (same as for the leaf samples) and transferred in an icebox to the Lab. They were sonicated in an ultrasonic cleaner (Transsonic 460 Elma, Partelli, Brescia, Italy) for 10 min, as described above, vortexed for 2 min and then the filters were removed. Buffer suspensions were centrifuged as above. Pellets were kept at −20 °C and were analysed as one sample per sampling day in DNA extraction.

### 2.3. DNA Extraction, Composition and Abundance of Microbial Communities

Total genomic DNA was extracted by using the PowerSoil DNA isolation kit from Qiagen Laboratories (Hilden, Germany) in accordance with the manufacturer’s instructions. The quantity of the DNA was between 5 and 10 ng μL^−1^, as measured by Nanodrop (ND-1000, Thermo Fisher Scientific, Waltham, MA, USA). The DNA samples were amplified using the bacterial-specific primers 785F (5′-GGATTAGATACCGTGGTA-3′) and 1185mR (5′-GAYTTGACGTCATCCM-3′), which target and amplify a final domain of around 415 bp of the 16S rRNA gene in theV4-V6 SSU region [36,37]. The PCR conditions were as follows: 94 °C for 3 min followed by 28 cycles at 94 °C for 30 s, 53 °C for 40 s and 72 °C for 1 min, which was followed by a final elongation step at 72 °C for 5 min. After amplification, PCR products were checked in 2% agarose gel to determine the success of amplification and the relative intensity of the bands. Subsequently, the PCR products were purified using calibrated Ampure XP beads (Beckman Coulter Life sciences, Brea, CA, USA) and the purified products were used to prepare the DNA libraries by following the Illumina MiSeq DNA high-throughput library preparation protocol. DNA library preparation and sequencing were performed at Mr. DNA (www.mrdnalab.com; Shallowater, TX, USA) on a MiSeq following the manufacturer’s guidelines. 

For the quantification of bacterial 16S rRNA genes in our samples, a primer set was used as previously described [38]. A serial of 10-fold dilutions of a recombinant plasmid containing a partial fragment of a bacterial 16S rRNA gene was used as external standard to perform the standard curve. The standard dilutions ranged from 10^4^ to 10^10^. The real-time PCR was performed in a LightCycler 480 (Roche Basel, Switzerland) instrument using the LightCycler 480 SYBR Green Master I (Roche, Basel, Switzerland)) following the manufacturer’s instructions. All samples, standards and negative controls were tested in triplicates. Finally, we used cycle threshold (CT) values to determine the number of 16S rRNA gene copies in our samples. 

### 2.4. Read Processing

The produced reads were processed using the *mothur* v1.34.0 software [39], following the standard operating procedure [40]. Briefly, forward and reverse reads were joined, and the barcodes were removed. Reads below 200 bp, with homopolymers higher than 8 bp and with ambiguous base calls, were removed from downstream analysis. The remaining reads were de-replicated to the unique sequences and aligned independently against SILVA 128 database, containing 1,719,541 bacterial SSU rRNA sequences [41]. Then, the reads suspected for being chimeras were removed using the UCHIME software [42]. Almost 25% of the reads were removed at this step. The remaining reads were clustered into operational taxonomic units (OTUs) at 97% similarity. To obtain a rigorous dataset, OTUs with a single read in the entire dataset were removed from the analysis as they were suspected of being erroneous sequences [43]; these corresponded to almost 50% of the OTUs. The resulting dataset was normalized to the lowest number of reads (5038 reads) with the subsample command in *mothur*. Taxonomic classification was assigned using SINA searches on the Silva 128 curated database [44]. After normalization, the reads belonging to OTUs affiliated with unclassified sequences at the domain level were removed in order to be confident that the produced dataset includes only bacterial reads. Chloroplast-related reads that were recovered were also removed from the dataset; there were no mitochondrial OTUs. Raw reads were submitted to GenBank-SRA under the accession number SRX3316528.

### 2.5. Data Analysis

We used the normalized dataset for all our analyses. Shannon, Simpson and Pielou’s evenness alpha-diversity estimators were calculated with the PAST 2.17c software [45]. The bacterial assemblages of the different samplings were compared using the Plymouth routines in the multivariate ecological research software package PRIMER v.6 [46]. The Bray–Curtis dissimilarity coefficients were calculated to develop the matrix based on OTU abundance in order to identify interrelationships between the samples and construct the cluster plots. For the determination of the OTUs responsible for the within and between group dissimilarities, the similarity percentage analysis (SIMPER) was applied [47]. 

We used paired t-tests to compare the summer and winter values of (i) the diversity indices of the epiphytic bacterial community, (ii) the richness (number of different OTUs belonging to bacteria) and abundance (bacterial 16S rRNA gene copies g^−1^ plant tissue) of the entire epiphytic bacterial community, and (iii) the richness and abundance of OTUs corresponding to the bacterial phyla and to the classes of the dominant Proteobacteria that were represented in the epiphytic community. For this analysis, we used Statistica 7 for Windows (StatSoft, Tulsa, USA).

OTUs were classified as abundant or rare in relation to their overall relative abundance, following HTS studies on prokaryotes [48,49,50]. Abundant OTUs were defined as those with relative abundance > 1%, whereas rare OTUs as those with abundance < 0.1% of the total number of reads in the entire dataset. As this classification operates at two levels, OTUs could be also distinguished as locally rare or locally abundant after the total number of reads per sample.

Furthermore, OTUs were classified as generalists and specialists based on Levins’ index [51]. Levins’ proposed that niche breadth could be estimated by measuring the individuals’ uniformity of distribution among the resource states. For this, specialization of each individual OTU was calculated according to Pandit et al. [52], using Levins’ niche width (*B_j_*) index [51] (1):

(1) $$B_{j}=\frac{1}{\sum_{i=1}^{N}p_{ij}^{2}}$$

where *pij* is the proportion of OTU *j* in sample *i*, and *N* is the total number of samples. Therefore, *B_j_* describes the extent of niche specialization based on the distribution of OTU abundances without taking into account the abiotic conditions in a local community. The values of the index range between 1 for singletons and a maximum value that varies depending on the dataset, which in our case was 15 (top generalist). OTUs with *B_j_* index higher than 10 were arbitrarily considered as generalists, while OTUs with *B_j_* lower than 5 as specialists [53].

For the network analysis, the relationship between OTUs was characterized through MINE statistics by computing the Maximal Information Coefficient (MIC) between each pair of OTUs, based on the number of reads of each OTU [54]. MIC captures correlations between data and provides a score between 0 (no correlation) and 1 (very strong correlation) that represents the strength of a relationship between data pairs. The sample pairings with MIC values > 0.5 (corresponding to *p*-values < 0.05) were used to visualize the strong correlations between OTUs [54]. The topological parameters of the respective networks were calculated with Cytoscape 3.0 [55]. Network Randomizer 1.1.2 [56] was used to generate random networks of the dataset in order to compare with and assess air and phyllosphere networks’ density.

## 3. Results

As determined by quantitative real time PCR, the number of bacterial 16S rRNA gene copies per gram of plant tissue of the Mediterranean phyllosphere studied was of the order of magnitude of 10^8^ and did not differ between seasons (Table 1). The number of bacterial 16S rRNA gene copies per cubic meter of air was of the order of 10^6^. Values for microbial abundance in the air and in the phyllosphere cannot be directly compared as the units differ—i.e., in number of copies per unit of weight, in the case of the phyllosphere, and per unit of volume, in the case of the air. Nevertheless, taking into consideration that the density of air at sea level is approximately 1/800th the density of water, what corresponds to 1.25 kg m^−3^ [57], we can estimate the microbial abundance in the air on a per unit of weight basis (Table 1). Values are of the order of 10^3^ per gram of air. 

After read processing, the removal of singletons, non-bacterial sequences and normalization, a total of 890 different OTUs were detected. Rarefaction curves that were calculated for all samples (Supplement Figure S1) indicate that a satisfying part of the diversity was recovered with the sequencing effort applied in most samples. There were 771 OTUs in the overall summer dataset and 430 in the winter one. Of these, 750 and 420 OTUs, respectively, corresponded to the phyllosphere. Air samples had far lower numbers of OTUs: 128 in summer and 86 in winter. The Shannon diversity index of the epiphytic community, estimated after the OTU abundances, was higher in summer (3.63 ± 0.18) than in winter (2.95 ± 0.15). Similarly, the Pielou’s evenness index was higher in summer (0.68 ± 0.02 vs. 0.59 ± 0.03), but the Simpson diversity index did not differ between seasons (Table 1). On average, there were 233.8 ± 22.4 OTUs per phyllosphere sample in summer, whereas 146.0 ± 10.9 in winter (Table 1). Summary results regarding the different OTUs in leaf and air samples are given in Table 2.

**Figure 1:**
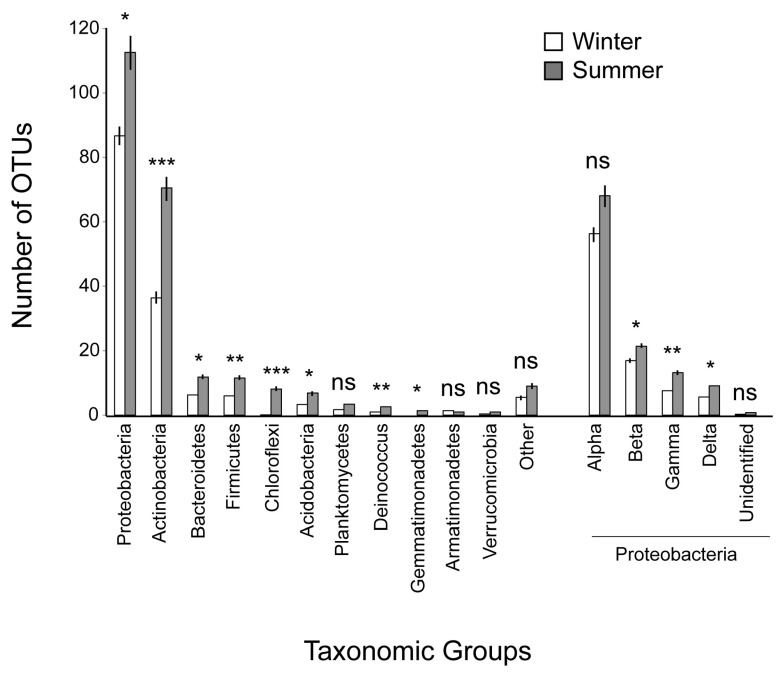
Number of OTUs belonging to the major high-level taxonomic groups of bacteria that were represented in the microbial community of the Mediterranean phyllosphere examined, in summer and winter, and paired t-test results. Under ‘Other’, Latescibacteria, Nitrospirae, Saccharibacteria, Spirochaetae, the WD272 group and unknown bacteria are included. For each parameter, the mean values and their standard errors are given; *: *p* < 0.05; **: *p* < 0.01; ***: *p* < 0.001; ns: non-significant.

Proteobacteria was the prominent phylum in the bacterial community of the Mediterranean phyllosphere examined (Table 3), followed by Actinobacteria. Bacteroidetes, Firmicutes, Acidobacteria, Deinococcus, Chloroflexi and Planctomycetes had a considerable participation, though two to three orders of magnitude lower than that of the previous two phyla. Other participating phyla were Gemmatimonadetes, Armatimonadetes, Verrucomicrobia and also Latescibacteria, Nitrospirae, Saccharibacteria, Spirochaetae and the WD272 group; there were also a few unidentified bacteria. Within Proteobacteria, Alphaproteobacteria was the dominant class, followed by Betaproteobacteria. The rank at the phylum and class levels remained quite similar if instead of OTU abundance, the number of different OTUs representing each of the above taxa (OTU richness) was taken into consideration (Figure 1). 

At a lower taxonomic level, Rhizobiales was the bacterial order represented in the phyllosphere by the highest number of OTUs (89) (Table 4). Rhodospirillales, Micrococcales, Sphingomonadales, and Burkholderiales followed, all with similar number of different OTUs (47–57). Rhizobiales was first in rank in the air, too, but represented by a far lower number of OTUs (18); again Burkholderiales, Sphingomonadales and Micrococcales followed, represented by 12–16 OTUs. There were also striking differences. Representatives of Acidimicrobiales and Planktomycetales, with a high participation in the phyllosphere (18 and 16 OTUs, respectively), were absent in the air, whereas other major taxa were at a far lower rank in the air than in the phyllosphere and vice versa (Table 4).

The most abundant OTU in the phyllosphere was closely related to a *Sphingomonas* strain. Of the next four most abundant OTUs, one was closely related to another *Sphingomonas* strain, one to a *Methylobacterium* strain and the other two to Rhizobiales and Burkholderiales strains.

**Figure 2:**
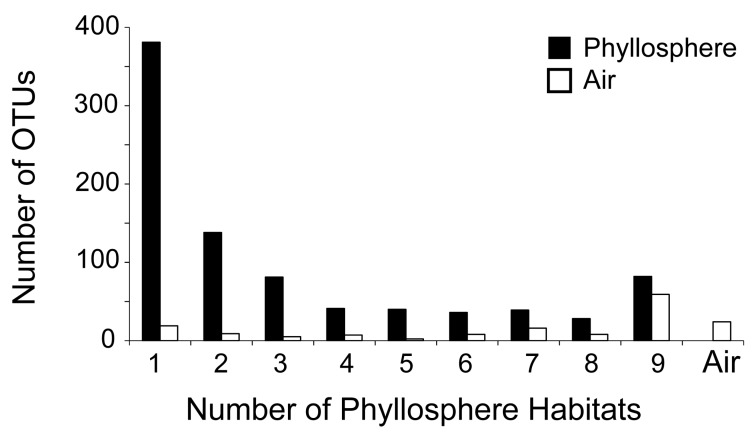
Number of bacterial OTUs in the phyllosphere of a Mediterranean ecosystem that were detected in only one up to all nine phyllosphere habitats (species) that were sampled, and number of these OTUs that were also found in the air; the final bar marked as ‘Air’ corresponds to the OTUs that were not detected in any phyllosphere habitat but only in the air. Numbers are determined from the entire dataset, with summer and winter data combined.

The vast majority of OTUs (89%) in the whole database were categorized as rare. Only 15 OTUs were categorized as abundant (Table 2 and Table 5); these were present in all phyllosphere habitats and in the air and were all locally abundant in one and up to four habitats. There were 82 universal OTUs that were detected in all phyllosphere habitats (Figure 2). Corresponding to 9% of all recovered OTUs, these universal OTUs belong to Proteobacteria, Actinobacteria, Bacteroidetes, Firmicutes and Acidobacteria. Markedly higher was the number of OTUs (381) that were found in only one phyllosphere habitat (Figure 2). These narrow-niche OTUs made 44% of the phyllosphere total (Table 2). 

According to the Levins’ index, the vast majority of OTUs (89%) were specialists, with only 1% being generalists. All 10 generalist OTUs belong to Alphaproteobacteria and Actinobacteria; these were also among the 82 universal OTUs and, with the exception of one, they were also present in the air (Table 5). Of the specialist OTUs, six were abundant. None of the members of Alphaproteobacteria, a major bacterial class in the Mediterranean epiphytic community both in terms of OTU richness and OTU abundance, participated in the group of abundant specialists. 

There was a pronounced seasonal difference in the structure of the phyllosphere microbial community. OTU richness was higher in summer for the entire community (Table 1), for all major bacterial phyla, i.e., Proteobacteria Actinobacteria, Bacteroidetes, Firmicutes, Acidobacteria, and Chloroflexi, for other minor ones, such as Deinococcus and Gemmatimonadetes, and also for the Proteobacteria classes, except for the dominant Alphaproteobacteria that did not differ between seasons (Figure 1). For the taxa differing between seasons in terms of OTU richness, the summer value was in general one to three times higher than the winter value, but Chloroflexi was essentially a summer taxon; all but one of the Chloroflexi OTUs were detected in summer. Gemmatimonadetes was similarly a summer taxon but it was represented by far fewer OTUs than Chloroflexi. In terms of OTU abundance, there was no difference between seasons of the phyla represented, except for Chloroflexi, Gemmatimonadetes and Firmicutes that were more abundant in summer. Also, the class of Betaproteobacteria was more abundant in summer, whereas that of Alphaproteobacteria in winter (Table 3). In summer, there was also a higher number of OTUs with exclusive occurrence in only one phyllosphere habitat (Table 1). 

**Figure 3:**
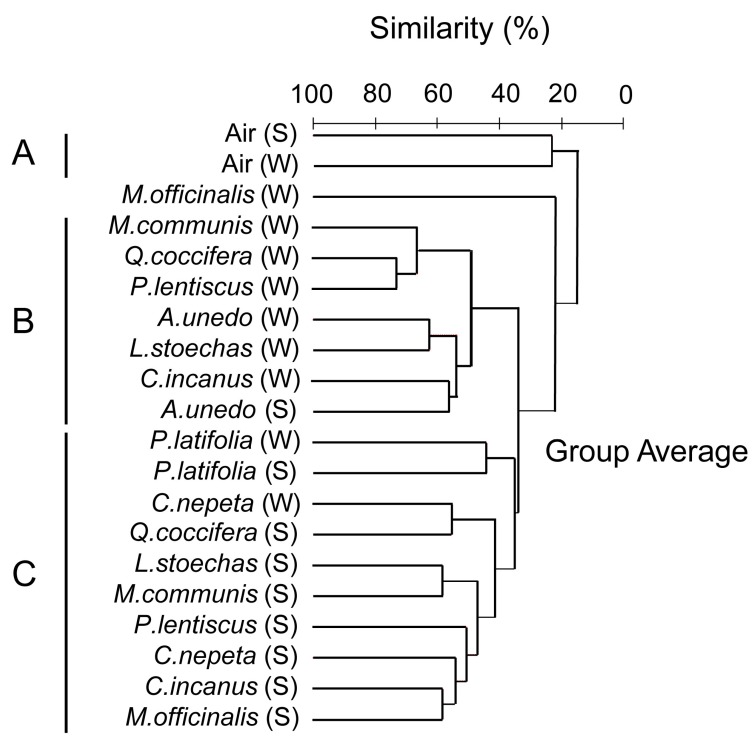
Cluster diagram based on Bray–Curtis dissimilarities calculated according to the number of reads of bacterial OTUs that were detected in the phyllosphere and in the air of the Mediterranean ecosystem studied; (S) represents summer samples and (W) winter samples. Three major clusters are formed: cluster A is made of air samples, whereas B and C are made of phyllosphere samples, primarily winter and summer ones, respectively.

**Figure 4:**
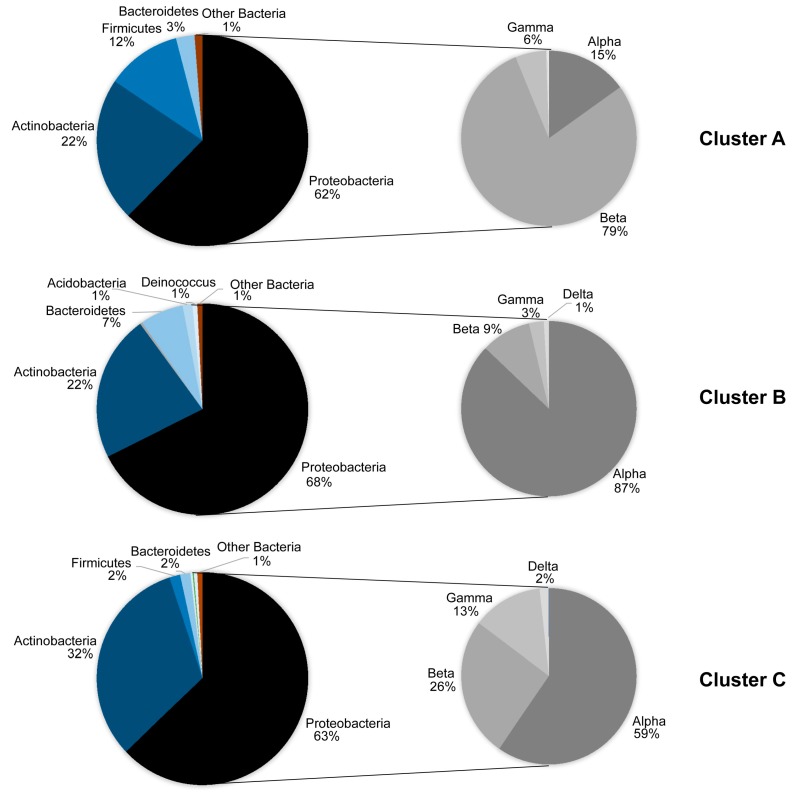
Relative abundances of the major high-level taxonomic groups of bacteria in the three clusters of Figure 3 that are formed using Bray–Curtis dissimilarities and on the basis of SILVA 128 database. To facilitate reading, the groups indicated have >1% of the total number of reads. Cluster A is made of air samples, whereas clusters B and C are made of phyllosphere samples, primarily winter and summer ones, respectively.

Given that our sampling did not allow direct comparisons between the phyllosphere and the airborne microbial communities, we subjected our samples to Cluster analysis, according to Bray–Curtis dissimilarities. Three major clusters emerged at >20% level of similarity (Figure 3). Cluster A consisted of the two air samples. Clusters B and C, separated at a 35% level of similarity, consisted of phyllosphere samples, primarily of winter and summer, respectively. The relative participation of the phyla and Proteobacteria classes in each of these three clusters is shown in Figure 4. Bacteria that do not belong to Proteobacteria, Actinobacteria, Bacteroidetes and Firmicutes had a very low contribution in the air, less than 1%, and whereas Alphaproteobacteria was the dominant Proteobacteria class in the phyllosphere, Betaproteobacteria was the dominant one in the air. Also, Actinobacteria had a far higher relative participation in the summer phyllosphere samples.

The SIMPER analysis, based on within cluster similarities, indicated the assemblages of OTUs that were responsible for the formation of each cluster: 17 OTUs for cluster A, 24 for cluster B, and 44 for cluster C (Supplement Table S2). Seven OTUs were responsible solely for the formation of cluster A. Of these, three belonged to Alphaproteobacteria, two to Firmicutes and the remaining two to unknown strains that had been reported to be isolated from the soil environment. For the formation of cluster B, three OTUs were solely responsible, of which two belonged to Alphaproteobacteria and one to Acidobacteria. For the formation of cluster C, 19 OTUs were solely responsible, with > 50% of them belonging to Actinobacteria strains (Supplement Table S2).

**Figure 5:**
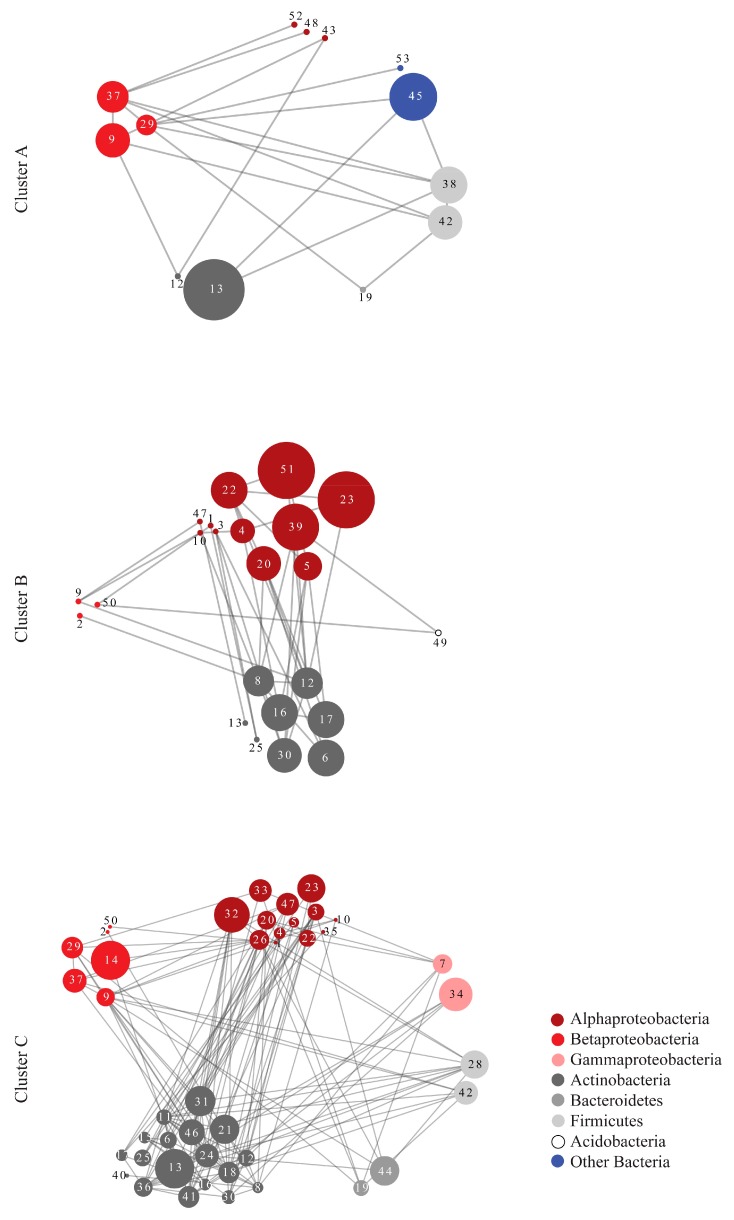
Networks of Maximal Information Coefficient (MIC) correlations (edges) based on the abundance of the OTUs (nodes) responsible for the formation of the three clusters in Figure 3, according to SIMPER analysis. The different colors represent different taxonomic groups. The numbers within each node correspond to the serial number of each OTU in Supplement Table S2. The size of the nodes is analogous to the clustering coefficient of each OTU with larger nodes representing key OTUs for the network, in terms of connectivity and centrality.

The correlations of the OTUs responsible for the formation of each cluster were calculated according to Maximal Information Coefficient (MIC) values. Network analysis was implemented on the OTUs exhibiting strong positive correlations (high MIC values, corresponding to a *p*-value < 0.05) in each cluster (Figure 5). Even though there are no physical interactions among OTUs found on the leaves of different host plants, network analysis provides indications of similar or opposing trends in OTU occurrence patterns. From the topological parameters calculated for each network, it was evident that the microbial community associated with cluster C was the most connected of the three. In particular, the clustering coefficient, the number of shortest paths, and the average number of neighbors took the highest values in cluster C (Table 6).

Network analysis also provided a visualization of the OTU correlations, indicating that different taxonomic groups had strong correlations both within and among them in each cluster. In cluster B, consisting mainly of winter samples, Alphaproteobacteria showed the highest number of correlations followed by Actinobacteria (Figure 5). In cluster C, consisting mainly of summer samples, Actinobacteria presented the highest number of correlations, while Betaproteobacteria, with a negligible role in the network structure for the other two clusters, presented high connectivity here. The topological parameters of the networks suggest that cluster C has a denser network structure than the other clusters: the Clustering Coefficient and the Average Number of Neighbors were higher in the network of cluster C (Table 6). Furthermore, topological parameters, such as the Clustering Coefficient, Centralization, and Heterogeneity, were approximately 2.5, 2 and 1.8 times higher, respectively, for the observed cluster C network relative to a random network of the same size suggesting indeed a denser structure than expected by chance alone [58].

## 4. Discussion

The study of the phyllosphere microbial community in a Mediterranean ecosystem showed that Proteobacteria is the dominant phylum in terms of both richness and abundance and that this holds true irrespective of season. This is also true for the airborne microbial community. However, the relative abundance in terms of average number of reads of bacterial classes within Proteobacteria does not remain constant between seasons. The contribution of the dominant Alphaproteobacteria decreased in summer, whereas that of Betaproteobacteria increased.

Results regarding the composition of the microbial community of the Mediterranean phyllosphere present similarities with reports from other ecosystem types of the world with different assemblages of plant species. Proteobacteria is most often the dominant group, with a participation ranging from less than 40% in tropical [4] to more than 80% in temperate forests [28,29], on floating macrophytes [59] or in the phyllosphere of individual crop species, such as *Spinacea oleracea* [60]. Depending on the conditions prevailing, Actinobacteria, Acidobacteria, Firmicutes, Bacteriodetes are reported as second in rank phyla.

Two *Sphingomonas-* and one *Methylobacterium*-related OTUs were amongst the five most abundant OTUs in the Mediterranean phyllosphere. Epiphytic microbial populations of soybean, clover and *Arabidopsis* [14] are also reported to be largely composed of *Sphingomonas* and *Methylobacterium*, and a *Sphingomonas* strain was among the five most abundant strains in the tree phyllosphere of the Brazilian Atlantic forest [27]. The dominance in the phyllosphere of methylotrophic and other taxa consuming one-carbon compounds, or of enzymes involved in the related metabolic reactions is also reported in a number of other cases [4,5,18,25,61] and has been associated with degradation processes of compounds that are toxic to plants, to humans or to the environment that are carried out by epiphytic microorganisms [5]. Dominance of these taxa in different plants, ecosystem types or geographical areas suggests major advantages from this type of symbiosis, which needs to be further explored.

It is reported that the phyllosphere microbial communities are very diverse in terms of richness, but not so in terms of evenness and that, generally, they comprise a few very well represented taxa and a large number of very rare ones [62]. This is also the case for the Mediterranean phyllosphere that we studied. Only 2% of the phyllosphere OTUs were identified as abundant, whereas 87% were identified as rare. Furthermore, the microbial community in this Mediterranean ecosystem is made up almost exclusively of habitat specialists with only very few habitat generalists.

A comparison of the epiphytic community with the airborne inoculum reveals large differences. Expressed on a per gram basis, the size of the airborne microbial community is approximately five orders of magnitude lower than that of the phyllosphere. This much thinner community bears far fewer taxa than those colonizing the phyllosphere, whereas microbes in abundance in the phyllosphere are not present in the air at the time of sampling and vice versa. A number of reasons can explain the differences between the two communities. For instance, taking air samples for a limited amount of time may not suffice to capture the full diversity of the airborne community; or, the sensitivity of the method does not allow detection at very low abundance. However, air is only a medium of microbial transfer constantly inoculating leaves with a range of taxa from various sources [63,64,65]. Microbes may land on leaves but then they are sorted out [2,66]. The epiphytic microbial community should not necessarily mirror the airborne inoculum at the time of sampling. Inocula and selection processes of the past play major roles in determining its structure.

With culture-dependent methods, it was found that the marked seasonality of the Mediterranean climate is not reflected in the size of the epiphytic microbial community [32]. Similar is the result with the culture-independent method that we used: the level of colonization did not differ between summer and winter. This was also true for Proteobacteria and Actinobacteria, which make the largest part of the community, and for several other phyla, but not for Firmicutes, Gemmatimonadetes and Chroroflexi, and the major Proteobacteria classes. In contrast, there was a seasonal effect on richness. This was higher in summer for the entire bacterial community and for most of the taxonomic groups examined, with none of the other taxa showing higher values in winter. This higher summer richness did not always lead to higher values of the diversity indices. The Pielou’s evenness index was higher in summer suggesting a more homogenous OTU distribution in this season compared to winter. The Shannon diversity index differed also between seasons (higher in summer) but the Simpson index did not. This is explained by the fact that the Simpson index puts very little weight to rare species, which are numerous in the community, particularly in summer. 

Experimental evidence suggests that the richness of the epiphytic bacterial community responds to climatic stressors and that it is very much influenced by drought [31]. The epiphytic microbial community of the Mediterranean phyllosphere that we studied is clearly richer in summer. This combined with the fact that the overall abundance does not change in summer, although the relative abundance of the dominant Alphaproteobacteria falls, suggest that the stressful summer conditions affect primarily the dominant members of the community. The relaxation of communities from extreme dominance of some of its members gives the opportunity to others to establish. This could be the case of Chloroflexi, which seems to be a typically summer phylum in the Mediterranean phyllosphere, although its representatives have been reported from various environments including anaerobic [67,68] and high-mountain ones [69].

The highest number of co-occurring/co-abundant OTUs that were detected through network analysis in winter corresponds mainly to Alphaproteobacteria, whereas in summer to Actinobacteria. Betaproteobacteria, with a negligible role in the winter phyllosphere network, increased considerably their co-occurrence patterns in summer. This change is in accordance with the increase in Betaproteobacteria in both richness and abundance and suggests an important role of this class in community structure in summer. Additionally, the summer network exhibits denser inter-associations among the correlated OTUs, as shown by the topological parameters calculated for this network compared to those for the winter and random networks. It has been suggested that robustness can increase with higher connectivity and denser taxa interactions, independent of taxa abundance [70], in the same way that increased taxa richness can stabilize community structure against environmental changes [71]. 

This is not the first time that season is found to play an important role in determining the composition of the phyllosphere bacterial community. Rastogi et al. [22] reported Proteobacteria, Firmicutes, Bacteroidetes and Actinobacteria to be the most abundantly represented phyla on lettuce foliage, as we found for the Mediterranean phyllosphere community, and a clear separation between winter and summer samples. In another study of a single *Magnolia grandiflora* tree, in which sampling took place at four seasons in one year and repeatedly at one season for three years [25], great temporal changes were detected, with the summer leaf community being very distinct. These temporal changes were not only seasonal; communities sampled at the same time in different years showed considerable differences, what made authors argue that seasonal patterns may not be predictable from year to year.

Knowledge of the structure of the epiphytic microbial communities in different environments and how these change with time will contribute to answering open questions on the specific functions of the microbiome on plant leaves and of the specific benefits of the partnership [72]. We found the microbial community of the Mediterranean phyllosphere to differ considerably from the air inoculum at the time of sampling, indicating selection by plants of the microbial community to be established on their leaves. This epiphytic community is dominated by habitat specialists and becomes much richer, more distinct (according to the number of OTUs on a single sample), even (according to the Pielou’s eveness index) and diverse (according to the Shannon index), denser and more connected (according to network analysis) in summer. These summer features of the epiphytic microbial community could be considered as contributing to community stability under the adverse, hot and dry conditions of the Mediterranean summer, whereas the similarity in bacterial abundance in both seasons suggests that resources may not suffice for much higher population sizes.

## Figures and Tables

**Table 1:** Paired t-test results for bacterial 16S rRNA gene copies, number of bacterial operational taxonomic units (OTUs) per sample, number of OTUs present in only one sample, and Simpson, Shannon and Pielou’s Evenness diversity indices estimated after the OTUs present, in summer and winter, for the phyllosphere bacterial community of the Mediterranean ecosystem studied. For each parameter, the mean values and their standard errors are given; d.f.: degrees of freedom; *: *p* < 0.05; **: *p* < 0.01; ns: non-significant. The numbers of bacterial 16S rRNA gene copies per m^3^ air and per gram of air are also given. For air values, no t-test could be conducted.

Attribute	Summer	Winter	d.f.	*t*-Value	*p*-Value	
Bacterial 16S rRNA gene copies g^−1^ plant tissue	2.3 ± 0.52 (× 10^8^)	8.8 ± 4.32 (× 10^8^)	8	−1.57	0.15	ns
Number of OTUs per sample (OTU richness)	233.8 ± 22.39	146.0 ± 10.85	8	3.91	0.004	**
Number of OTUs in only one sample	30.4 ± 5.62	8.9 ± 1.52	8	3.48	0.01	**
Simpson (*1-D*) index	0.9 ± 0.02	0.9 ± 0.02	8	1.40	0.19	ns
Shannon (*H*) index	3.6 ± 0.18	2.9 ± 0.15	8	2.44	0.04	*
Pielou’s Evenness (*J*) index	0.68 ± 0.02	0.59 ± 0.03	8	2.67	0.01	**
Bacterial 16S rRNA gene copies m^−3^ air	2.45 × 10^6^	1.72 × 10^6^				
Bacterial 16S rRNA gene copies g^−1^ air	1.96 × 10^3^	1.38 × 10^3^				

**Table 2:** Summary results regarding the OTUs detected in the phyllosphere of the Mediterranean ecosystem studied and in the surrounding air.

Type of Bacterial OTUs	Number	Relative Overall Contribution (%)	Relative within Group Contribution (%)
OTUs in the database (after normalization)	890		
OΤUS in the phyllosphere	869	98	
OTUs in the air	157	18	
Abundant OTUs (>1% of all reads)	15	2	
Rare OTUs (<0.1% of all reads)	794	89	
Generalist OTUs based on the Levins’ index	10	1	
Specialist OTUs based on the Levins’ index	788	89	
OTUs on leaves in summer	750	84	86 ^1^
OTUs on leaves in winter	420	47	48 ^1^
OTUs in the air in summer	128	14	81 ^2^
OTUs in the air in winter	86	10	55 ^2^
Abundant OTUs on leaves (>1% of all reads)	14	2	2 ^1^
Rare OTUs on leaves (<0.1% of all reads)	770	87	89 ^1^
OTUs on all habitats (species) (universal OTUs)	82	9	9 ^1^
OTUs on only one habitat (species)	381	43	44 ^1^

^1^ After the number of OTUs detected in the phyllosphere; ^2^ after the number of OTUs detected in the air.

**Table 3:** Paired t-test results for OTU abundance between summer and winter for the major high-level taxonomic groups of bacteria represented in the phyllosphere bacterial community of the Mediterranean ecosystem studied. Under ‘Other’, Latescibacteria, Nitrospirae, Saccharibacteria, Spirochaetae, the WD272 group and unknown bacteria are included. For Gemmatimonadetes, with zero values in winter, we tested whether summer values differed on average significantly from 0. For each bacterial group compared, the mean values and their standard errors are given; d.f.: degrees of freedom; *: *p* < 0.05; **: *p* < 0.01; ns: non-significant.

Bacterial Group	OTU Abundance					
	Summer	Winter	df	*t*-Value	*p*-Value	
Proteobacteria	2870.9 ± 218.80	3371.9 ± 290.7	8	−1.54	0.16	ns
Actinobacteria	1543.8 ± 160.20	1126.6 ± 272.2	8	1.59	0.149	ns
Firmicutes	87.2 ± 22.47	17.0 ± 4.4	8	3.12	0.014	*
Bacteroidetes	80.9 ± 11.63	286.2 ± 257.1	8	−0.789	0.45	ns
Acidobacteria	18.2 ± 4.81	62.3 ± 20.2	8	−2.23	0.055	ns
Deinococcus	34.6 ± 24.14	7.6 ± 4.8	8	1.12	0.29	ns
Chloroflexi	29.1 ± 7.57	0.1 ± 0.1	8	3.82	0.005	**
Planctomycetes	7.8 ± 1.31	10.6 ± 6.5	8	−0.41	0.69	ns
Gemmatimonadetes	2.3 ± 0.85	0	8	2.55	0.03	*
Armatimonadetes	0.2 ± 0.24	0.6 ± 0.5	8	−0.8	0.44	ns
Verrucomicrobia	0.1 ± 0.11	0.2 ± 0.2	8	−1	0.35	ns
Other	66.9 ± 24.73	31.9 ± 11.1	8	1.41	0.19	ns
*Proteobacteria classes*						
Alphaproteobacteria	1750.7 ± 167.37	2761.7 ± 257.5	8	−3.39	0.009	**
Betaproteobacteria	759.4 ± 135.77	369.8 ± 120.9	8	2.16	0.046	*
Gammaproteobacteria	304.7 ± 157.91	212.0 ± 102.3	8	0.94	0.37	ns
Deltaproteobacteria	54.8 ± 12.72	28.3 ± 9.2	8	2.064	0.07	ns
Unidentified	1.3 ± 0.48	0.1 ± 0.1	8	2.81	0.022	*

**Table 4:** The first fifteen orders of phyllosphere bacteria ranked in descending order by the number of OTUs detected in the phyllosphere of the Mediterranean ecosystem studied that belong to them. Given is also the representation of these orders in the surrounding air. Empty spaces under ‘Air’ mean that taxa other than the ones presented here had higher representation in the air.

Orders	Phyllosphere		Air	
	Number of OTUs	Rank by Number of OTUs	Number of OTUs	Rank by Number of OTUs
Rhizobiales	89	1	18	1
Rhodospirillales	57	2	6	8
Micrococcales	53	3	12	4
Sphingomonadales	48	4	14	3
Burkholderiales	47	5	16	2
Propionibacteriales	44	6	10	6
Rhodobacterales	36	7	3	12
Myxococcales	25	8	1	
Bacillales	25	8	7	7
Cytophagales	24	10	2	
Flavobacteriales	22	11	11	5
Frankiales	21	12	5	9
Acidimicrobiales	18	13	0	
Plantkomycetales	16	14	0	
Caulobacterales	14	15	2	
Corynebacteriales	14	15	4	10
Enterobacteriales	14	15	4	10

**Table 5:** Abundant (making >1% of the total number of reads) and universal OTUs (present in all phyllosphere habitats) that are assessed as generalists according to Levins’ index and their closest relatives. The symbol ‘**+**’ represents presence in the sample, whereas ‘**√**’ represents local abundance (>1% of the reads of a sample). Among the abundant OTUs, marked with * are the specialists according to Levins’ index. For further details about the closest relatives of the OTUs detected, see Supplement Table S2.

OTUs	Putative Affiliation	Presence and Local Abundance in the Air and the Phyllosphere Samples									
		Air	*Quercus coccifera*	*Arbutus unedo*	*Phillyrea latifolia*	*Pistacia lentiscus*	*Myrtus communis*	*Calamintha nepeta*	*Cistus incanus*	*Lavandula stoechas*	*Melissa officinalis*
**Abundant**											
OTU003	Alphaproteobacteria *Methylobacterium* sp.1	**+**	**+**	**+**	**+**	**+**	**+**	**√**	**+**	**+**	**+**
OTU004	Alphaproteobacteria *Sphingomonas faeni*	**+**	**+**	**+**	**+**	**+**	**+**	**+**	**+**	**+**	**√**
OTU005	Alphaproteobacteria Rhizobiales	**+**	**+**	**√**	**+**	**+**	**√**	**+**	**√**	**√**	**+**
OTU007 *	Gammaproteobacteria *Pseudomonas* sp.	**+**	**√**	**+**	**+**	**+**	**+**	**√**	**√**	**+**	**√**
OTU008	Actinobacteria *Friedmanniella* sp.	**+**	**+**	**+**	**+**	**√**	**+**	**+**	**+**	**+**	**+**
OTU009	Betaproteobacteria *Hydrogenophaga* sp.	**√**	**+**	**+**	**+**	**+**	**+**	**+**	**+**	**√**	**+**
OTU012	Actinobacteria *Amnibacterium* sp.	**+**	**√**	**+**	**+**	**+**	**+**	**+**	**+**	**+**	**+**
OTU013	Actinobacteria *Propionibacterium acne*	**√**	**+**	**+**	**+**	**+**	**+**	**+**	**+**	**√**	**+**
OTU020 *	Bacteroidetes *Chryseobacterium* sp.	**+**	**+**	**+**	**+**	**+**	**+**	**+**	**+**	**+**	**√**
OTU023	Alphaproteobacteria Rhizobiales	**+**	**+**	**+**	**+**	**+**	**√**	**+**	**+**	**+**	**+**
OTU024	Alphaproteobacteria *Methylobacterium* sp.2	**+**	**+**	**+**	**+**	**+**	**+**	**+**	**+**	**+**	**√**
OTU033 *	Betaproteobacteria *Ralstonia pickettii* S	**√**	**+**	**+**	**+**	**+**	**+**	**+**	**+**	**√**	**√**
OTU041 *	Gammaproteobacteria *Buchnera aphidicola*	**+**	**+**	**+**	**+**	**+**	**+**	**√**	**+**	**√**	**+**
OTU045 *	Actinobacteria *Kineococcus* sp.	**+**	**+**	**+**	**√**	**+**	**+**	**+**	**+**	**+**	**+**
OTU072 *	Firmicutes *Tumebacillus* sp.	**√**	**+**	**+**	**+**	**+**	**√**	**+**	**+**	**√**	**√**
**Universal OTUs, Generalists**											
OTU001	Alphaproteobacteria *Sphingomonas* sp.1	**+**	**+**	**+**	**+**	**+**	**+**	**+**	**+**	**+**	**+**
OTU003	Alphaproteobacteria *Methylobacterium* sp.1	**+**	**+**	**+**	**+**	**+**	**+**	**√**	**+**	**+**	**+**
OTU006	Actinobacteria *Frigoribacterium* sp.	**+**	**+**	**+**	**+**	**+**	**+**	**+**	**+**	**+**	**+**
OTU010	Alphaproteobacteria *Sphingomonas* sp.2	**+**	**+**	**+**	**+**	**+**	**+**	**+**	**+**	**+**	**+**
OTU016	Actinobacteria *Curtobacterium flaccumfaciens*	**+**	**+**	**+**	**+**	**+**	**+**	**+**	**+**	**+**	**+**
OTU021	Alphaproteobacteria Acetobacteraceae	**+**	**+**	**+**	**+**	**+**	**+**	**+**	**+**	**+**	**+**
OTU026	Actinobacteria *Kineococcus* sp.	**+**	**+**	**+**	**+**	**+**	**+**	**+**	**+**	**+**	**+**
OTU040	Alphaproteobacteria *Rhizobium* sp.	**+**	**+**	**+**	**+**	**+**	**+**	**+**	**+**	**+**	**+**
OTU042	Alphaproteobacteria *Roseomonas aerophila*	**+**	**+**	**+**	**+**	**+**	**+**	**+**	**+**	**+**	**+**
OTU152 ^#^	Alphaproteobacteria *Methylobacterium* sp.2 ^#^		**+**	**+**	**+**	**+**	**+**	**+**	**+**	**+**	**+**

^#^ OTU not included in Supplement Table S2; this is the closest relative with 99% similarity.

**Table 6:** Network topological parameters of the three clusters, performed by Cytoscape V.3.5., and the topological parameters of the respective random networks created by Network Randomizer 1.1.2.

Network Topological Parameters	Cluster A	Random Network A	Cluster B	Random Network B	Cluster C	Random Network C
Nodes	13	13	23	23	43	43
Edges (pairs)	20	20	40	40	142	142
Clustering coefficient	0.242	0.231	0.201	0.137	0.404	0.169
Connected components	1	1	1	2	1	1
Network diameter	4	5	7	5	6	4
Network radius	2	3	4	3	3	3
Network centralization	0.386	0.189	0.225	0.225	0.285	0.135
Shortest paths	156	156	506	462	1806	1806
Characteristic path length	2.051	2.231	2.763	2.333	2.408	2.163
Avg. number of neighbors	3.077	3.077	3.478	3.478	6.605	6.605
Network density	0.256	0.256	0.158	0.158	0.157	0.157
Network heterogeneity	0.617	0.412	0.508	0.555	0.619	0.332

## Supplementary Materials

The following are available online at https://www.mdpi.com/2076-2607/7/11/518/s1, Supplement Table S1. Weather conditions prevailing on the sampling days. Measurements on the spot were taken at the end of sampling. The other values of the meteorological parameters are from the weather station at Neos Marmaras, the nearest to the sampling site, at 13 km on a straight line, which is operated by the University of the Aegean in collaboration with the Forest Office of Polygyros and the National Observatory of Athens. Supplement Table S2. OTUs (54, in total) that are identified as responsible for the within cluster similarities according to SIMPER analysis, their closest relative based on BLAST searches against SILVA 119 database, and the isolation source of the closest relative in that database. Supplement Figure S1. Rarefaction curves representing the number of OTUs against the number of high-quality reads.
