# Supplementary material for: Metagenomic Characterization Reveals Pronounced Seasonality in the Diversity and Structure of the Phyllosphere Bacterial Community in a Mediterranean Ecosystem
Source: Microorganisms. 2019 Nov 1;7(11):518. doi: 10.3390/microorganisms7110518 (PMC6920919; doi:10.3390/microorganisms7110518)
Supplement: Supplementary file 1 [file microorganisms-07-00518-s001.pdf]

microorganisms

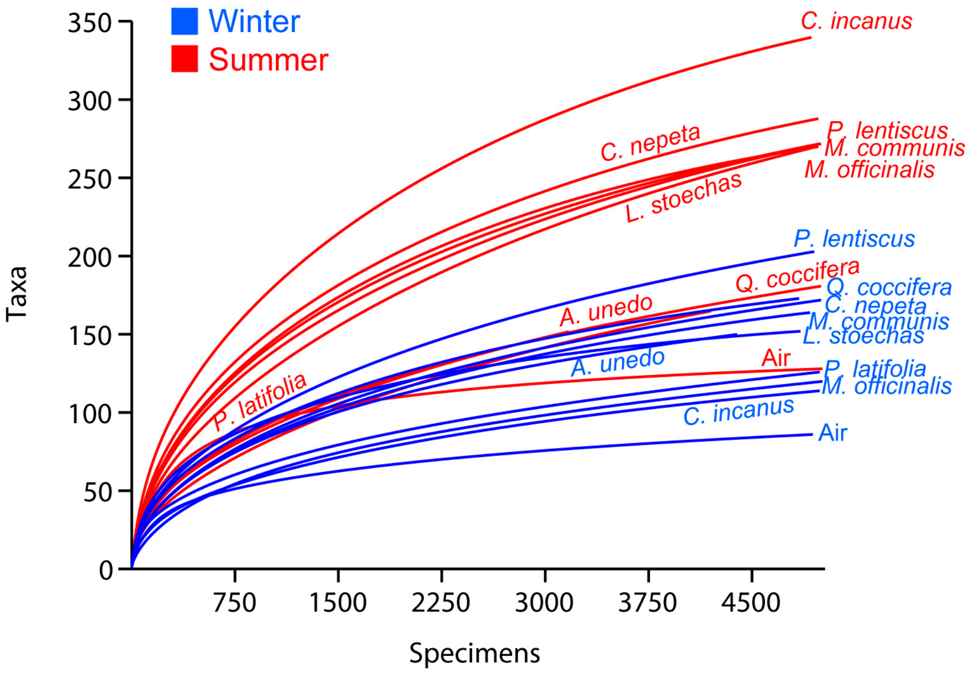

Supplement Figure S1. Rarefaction curves representing the number of OTUs against the number of high-quality reads.

Supplement Table S1. Weather conditions prevailing on the sampling days. Measurements on the spot were taken at the end of sampling. The other values of the meteorological parameters are from the weather station at Neos Marmaras, the nearest to the sampling site, at 13 km on a straight line, which is operated by the University of the Aegean in collaboration with the Forest Office of Polygyros and the National Observatory of Athens.

Measurements	Sampling Day	
	July 10, 2014	January 13, 2015
T (°C) on the spot	28.0	6.1
Average T (°C) for the day, weather station	27.7	7.1
Min T(°C) for the day, weather station	22.2	4.1
Max T(°C) for the day, weather station	33.8	10.4
Wind speed (km h^{-1}) on the spot	1.0	5.0
Average wind speed (km h^{-1}) for the day, weather station	4.5	5.5
Max wind speed (km h^{-1}) of the day, weather station	25.7	29.0
Rain (mm)	0	0

Supplement Table S2. OTUs (54, in total) that are identified as responsible for the within cluster similarities according to SIMPER analysis, their closest relative based on BLAST searches against SILVA 119 database, and the isolation source of the closest relative in that database.

OTUs	Putative Taxonomic Affiliation	Closest Relative (% Similarity) [NCBI Accession Number]	Isolation Source	Cluster A	Cluster B	Cluster C
1. OTU001	Alphaproteobacteria	*Sphingomonas* sp. (100%) [KJ652701]	Root hairs		√	√
2. OTU002	Betaproteobacteria	*Albidiferax* sp. (100%) [KF441631]	Water and sediment	√	√	√
3. OTU003	Alphaproteobacteria	*Methylobacterium* sp. (99%) [KP128697]	Spruce spider mite		√	√
4. OTU004	Alphaproteobacteria	*Sphingomonas faeni* (100%) [KT347530]	Nectar of *Linaria vulgaris*		√	√
5. OTU005	Alphaproteobacteria	Rhizobiales (99%) [KJ016001]	Lichen		√	√
6. OTU006	Actinobacteria	*Frigoribacterium* sp. (99%) [KR922126]	Cloud water	√	√	√
7. OTU007	Gammaproteobacteria	*Pseudomonas* sp. (100%) [GU000373]	Sediment			√
8. OTU008	Actinobacteria	*Friedmanniella* sp. (99%) [KR007619]	Polar oceanic water		√	√
9. OTU009	Betaproteobacteria	*Hydrogenophaga* sp. (100%) [KF287756]	Water from a copper mine	√	√	√
10. OTU010	Alphaproteobacteria	*Sphingomonas* sp. (99%) [KT443880]	DWTP filter effluent		√	√
11. OTU011	Actinobacteria	*Blastococcus* sp. (99%) [KM507603]	Deep sea			√
12. OTU012	Actinobacteria	*Amnibacterium* sp. (99%) [KM507591]	Deep sea	√	√	√
13. OTU013	Actinobacteria	*Propionibacterium acnes* (99%) [LN998080]	Stool specimen	√	√	√
14. OTU014	Betaproteobacteria	Oxalobacteraceae sp. (99%) [KM187416]	Amphibian			√
15. OTU015	Actinobacteria	*Actinoplanes* sp. (99%) [LC027115]	Leaf litter			√
16. OTU016	Actinobacteria	*Curtobacterium flaccumfaciens* (100%) [LN997873]	Milking machine biofilm		√	√
17. OTU017	Actinobacteria	*Actinomycetospora* sp. (99%) [GQ494028]	Culture strain		√	√
18. OTU019	Actinobacteria	*Geodermatophilus* sp. (100%) [LN626270]	Limestone			√
19. OTU020	Bacteroidetes	*Chryseobacterium* sp. (99%) [KR233779]	Surface soil of a glacier	√		√
20. OTU021	Alphaproteobacteria	Acetobacteraceae sp. (96%) [KJ606803]	Antarctic lichen		√	√
21. OTU022	Actinobacteria	*Geodermatophilus* sp. (100%) [KR184573]	Rhizosphere soil			√
22. OTU023	Alphaproteobacteria	Rhizobiales (100%) [JF814885]	Lichenized fungi		√	√
23. OTU024	Alphaproteobacteria	*Methylobacterium* sp. (100%) [KT380689]	Culture strain		√	√
24. OTU025	Actinobacteria	*Nocardioides* sp. (99%) [KJ191041]	Sediment			√
25. OTU026	Actinobacteria	*Kineococcus* sp. (100%) [KR265715]	Culture strain	√	√	√
26. OTU028	Alphaproteobacteria	*Paracoccus* sp. (99%) [KJ191092]	Sediment			√
27. OTU031	Betaproteobacteria	*Massilia* sp. (99%) [KF681058]	Soil		√	√
28. OTU032	Firmicutes	*Planomicrobium soli* (99%) [NR_134133]	Soil			√
29. OTU033	Betaproteobacteria	*Ralstonia pickettii* (100%) [KT444584]	Rhizosphere of rice	√		√
30. OTU034	Actinobacteria	*Nakamurella panacisegetis* (100%) [NR_108869]	Soil of ginseng field		√	√
31. OTU036	Actinobacteria	*Cellulomonas* sp. (99%) [KM507609]	Deep sea			√
32. OTU038	Alphaproteobacteria	*Craurococcus roseus* (95%) [LN90785]	Culture strain			√

33. OTU040	Alphaproteobacteria	*Rhizobium* sp. (99%) [KJ191012]	Sediment			√
34. OTU041	Gammaproteobacteria	*Buchnera aphidicola* (99%) [JX998123]	Culture strain			√
35. OTU042	Alphaproteobacteria	*Roseomonas aerophila* (99%) [KR364889]	Stem			√
36. OTU045	Actinobacteria	*Kineococcus* sp. (99%) [KM507619]	Deep sea			√
37. OTU047	Betaproteobacteria	*Diaphorobacter* sp. (99%) [KP152654]	Coal	√		√
38. OTU050	Firmicutes	*Staphylococcus epidermidis* (100%) [KT390733]	Culture strain	√		
39. OTU055	Alphaproteobacteria	*Novosphingobium kunmingense* (98%) [NR_134106]	Culture strain		√	
40. OTU057	Actinobacteria	*Quadrisphaera granulorum* (99%) [AM887695]	Faeces			√
41. OTU067	Actinobacteria	Uncultured clone (100%) [HM444694]	Street dust			√
42. OTU072	Firmicutes	*Tumebacillus* sp. (100%) [KM882951]	Ice core	√		√
43. OTU074	Alphaproteobacteria	*Bradyrhizobium* sp. (100%) [LN876281]	Grassland	√		
44. OTU079	Bacteroidetes	*Epilithonimonas lactis* (99%) [KT767722]	Milk			√
45. OTU084	Unknown	Uncultured clone (100%) [AB991511]	Annelid worm	√		
46. OTU093	Actinobacteria	*Kocuria* sp. (99%) [KU560441]	Culture strain			√
47. OTU099	Alphaproteobacteria	*Novosphingobium fluoreni* (99%) [KT719953]	Spacecraft surfaces		√	√
48. OTU100	Alphaproteobacteria	*Bradyrhizobium* sp. (100%) [NR_074315]	Culture strain	√		
49. OTU113	Acidobacteria	*Granulicella* sp. (99%) [JX532055]	Pitcher plant fluid		√	
50. OTU120	Betaproteobacteria	*Comamonas* sp. (98%) [JF808874]	Sludge		√	√
51. OTU141	Alphaproteobacteria	*Neoasaia chiangmaiensis* (96%) [NR_113975]	Culture strain		√	
52. OTU166	Alphaproteobacteria	*Sphingobium* sp. (100%) [KU174187]	Contaminated soil	√		
53. OTU189	Unknown	Uncultured clone (100%) [KF107370]	Skin	√		
54. OTU203	Firmicutes	*Streptococcus salivarius* (100%) [CP014144]	Oral	√		
